# Rambles in Europe in 1839. With Sketches Eminent Surgeons, Physicians, &c.

**Published:** 1841-07-01

**Authors:** 


RAMBLES IN EUROPE, IN 1839. WITH SKETCHES OF PROMINENT SURGEONS, PHYSICIANS, MEDICAL SCHOOLS, &c. By *W. Gibson*, M.D. Professor of Surgery in the University of Pennsylvania, &c. Octavo. Philadelphia, 1841.

THESE light sketches—or "pencillings by the way"—are evidently by a practised hand—the emanations of a mind possessed of quick perception and facile delineation. Dr. Gibson seems to have been determined to furnish a strong contrast to some of our own tourists who travel from Niagara to New Orleans, their pens or pencils dipped in the bile that was stirred up by the transatlantic voyage. We will not assert that our good-natured confrere has not fallen a little beyond the—

Certi denique fines
Quos citra vel ultra nequeat consistere rectum, dipping his plume too often in the finest copal varnish, and diffusing the flattering unction rather too freely over every character with which he comes in contact. Certainly a more kind-hearted traveller and painter never traversed the old world from beyond the western wave. The Doctor was evidently predetermined to be pleased with everything—or at least every person—that met his eye on the banks of the Thames, the Forth, the Liffey, and the Seine. These likenesses, though almost always flattered, are never caricatured. The features and forms can never be mistaken, though the colouring is often higher than Nature acknowledges. Take, for example, the following excellent sketch of our departed friend—

SIR A. COOPER.

"Imagine a tall, elegantly formed man, moderately robust, with a remarkably pleasing and striking countenance, red, and fresh as a rose, apparently about fifty-eight or sixty years of age, but, in reality, above seventy, very agile and graceful in all his movements, simply, but handsomely attired, with the spirit and vivacity, and bearing of a youth, with, in short, no marks of advanced age, except a head as white as the driven snow,—and a very just conception may be formed of the appearance of Sir Astley Cooper." 17.

Sir B. Brodie's portrait is equally tranchant.

"His appearance was altogether different from what I had supposed; for, instead of being full, stout, and ruddy, as most Englishmen are, I found him thin, pale, and, seemingly, delicate and dyspeptic; the result, however, as it struck me, of hard professional work, mental as well as corporeal, rather than of natural feebleness of constitution. His countenance was pensive, and verging towards a melancholy cast, but the moment he spoke it was lighted up by a smile, so peculiarly winning and attractive, so strikingly benignant and intelligent, as (added to uncommon softness and sweetness of voice, with manners so gentle, unpretending and free from assurance or arrogance,) to be calculated, I thought, to captivate, irresistibly, the most fastidious taste." 22.

We can only make room for one or two more sketches.

Mr. Lawrence.

"I had not inquired about his personal appearance, and was, therefore, particularly struck, upon entering his study, with his fine, manly figure; his open, expressive, intelligent countenance; his large and well-proportioned head; his lofty and expanded forehead; his clear and brilliant complexion; his mild, but sparkling, gray eye: and then when he spoke in a tone so quiet, modest and unassuming, with a manner so gentle and conciliating, and expressed himself so kindly and affectionately towards our country—its institutions and citizens—I could not but feel I stood in the presence of a superior being, could almost imagine I had known him all my life, and warmed towards him insensibly, as if he had been an old, long-tried, and intimate friend." 26.

Mr. Guthrie.

"In outline, Mr. Guthrie's face resembles slightly that of the late Dr. Physick—his countenance is animated and expressive, and full of good humour and benevolence. He is, indeed, universally considered, I believe, to possess the most amiable feelings, but when roused by opposition, or cross-examined in courts of justice, is said to be so keen, searching, sarcastic and witty in his observations and replies, as to silence, in a short time, the most talented members of the bar. His stature is about the medium height—his form muscular, inclining to *embonpoint*, well turned, if not decidedly handsome, and his whole air and bearing lofty; but his manners, at the same time, so free, easy, engaging and devoid of affectation, as to gain, irresistibly, the confidence of strangers, and secure, in a short time, their attachment." 31.

Our author was agreeably disappointed with Mr. Liston, having heard that he was "eccentric, rough, and uncouth in his manners, and a perfect ursa major, upon whose humour there could be no dependence." We have known Mr. Liston a great many years, and certainly we formed a very different estimate of his character. Dr. Gibson soon found that he had been egregiously misinformed respecting this eminent surgeon.

"Such misrepresentations are, no doubt, to be traced, in some instances, to the apparent eccentricities of Mr. Liston; for though, seemingly, of robust frame and great strength of constitution, he is so solicitous of preserving his health, and is so confident of the value of active exercise on horseback, as for a long time to have kept hunters and a pack of hounds, which, while he lived at Edinburgh, he exercised at day-break, and long before most of his brethren were out of bed. It is said he has now abandoned the sport, having fractured his pelvis, and nearly broken his neck at some inordinate leap, and since that period has followed the exercise of a boatman on the Thames, by rowing every morning several miles before breakfast. He has a passion for domestic animals—horses, dogs, and cats. His enormous black cat, *Tom*, is almost as well known in London as Liston himself, being, not unfrequently, mounted alongside his master in the splendid chariot, and a constant guest at his hospitable board, where I had the honour of forming his acquaintance, by finding his foot in my soup before aware of its proximity to my plate." 35.

In this good-humoured way Dr. Gibson sketches most of the prominent medical characters in the three capitals of the British Isles, as well as in Paris—interweaving numerous sensible remarks on the various institutions which he examined. The work is very amusing, and will have a most extensive circulation, especially in the United States. This volume furnishes innumerable proofs of the talent, acquirements, and liberality of its author.

